# Supplementary material for: Pattern of Tick Aggregation on Mice: Larger Than Expected Distribution Tail Enhances the Spread of Tick-Borne Pathogens
Source: PLoS Comput Biol. 2014 Nov 13;10(11):e1003931. doi: 10.1371/journal.pcbi.1003931 (PMC4230730; doi:10.1371/journal.pcbi.1003931)
Supplement: Text S1 — Sensitivity analysis of distribution parameters on epidemic spreading. (PDF) [file pcbi.1003931.s001.pdf]

Pattern of tick aggregation on mice: larger than expected distribution tail enhances the spread of tick-borne pathogens: Supporting Information S1

Luca Ferreri*, Mario Giacobini, Paolo Bajardi, Luigi Bertolotti, Luca Bolzoni, Valentina Tagliapietra, Annapaola Rizzoli, Roberto Rosà
∗ E-mail: luca.ferreri@unito.it

S1 - Sensitivity Analysis of Distribution Parameters on Epidemic Spreading

In this section we explored the effect of parameters of tick burden distributions not at the best fit on the epidemic spreading. Results suggested that the larger the heterogeneity caused by parameters, the lower the epidemic threshold.

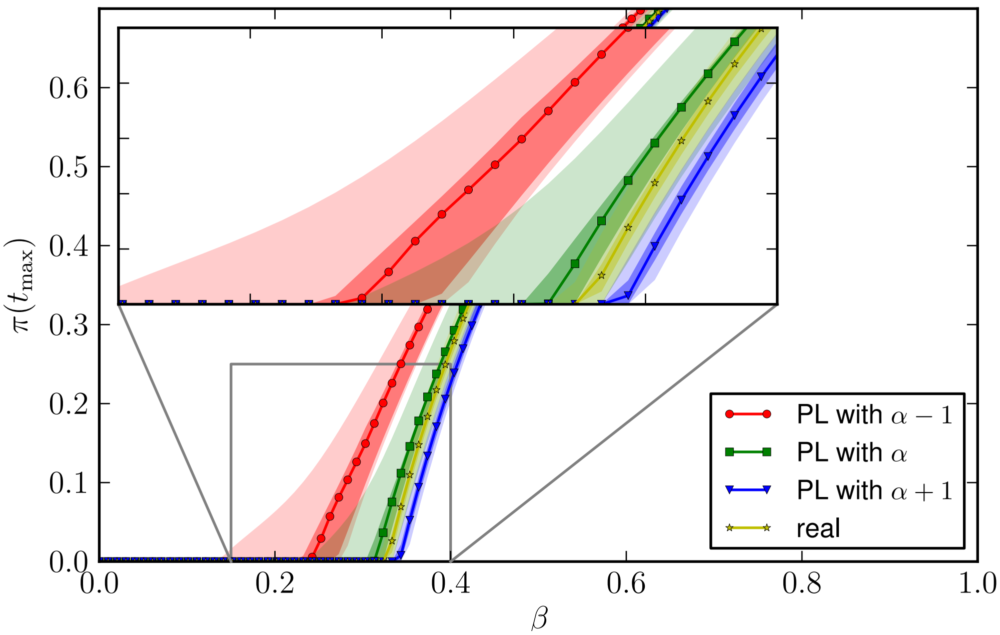

Figure 1. Median (lines), interquartile (darker areas) and 95% confidence intervals (lighter areas) of the final prevalence as a function of the transmission probability. Ticks burdens are described by PL distribution with different exponents (see legend). In particular, we explore the sensitivity of this function to variations in α, which represents the best fit parameter on the empirical data. The long-term prevalence obtained with tick burdens sampled from the empirical distribution is also plotted as benchmark.

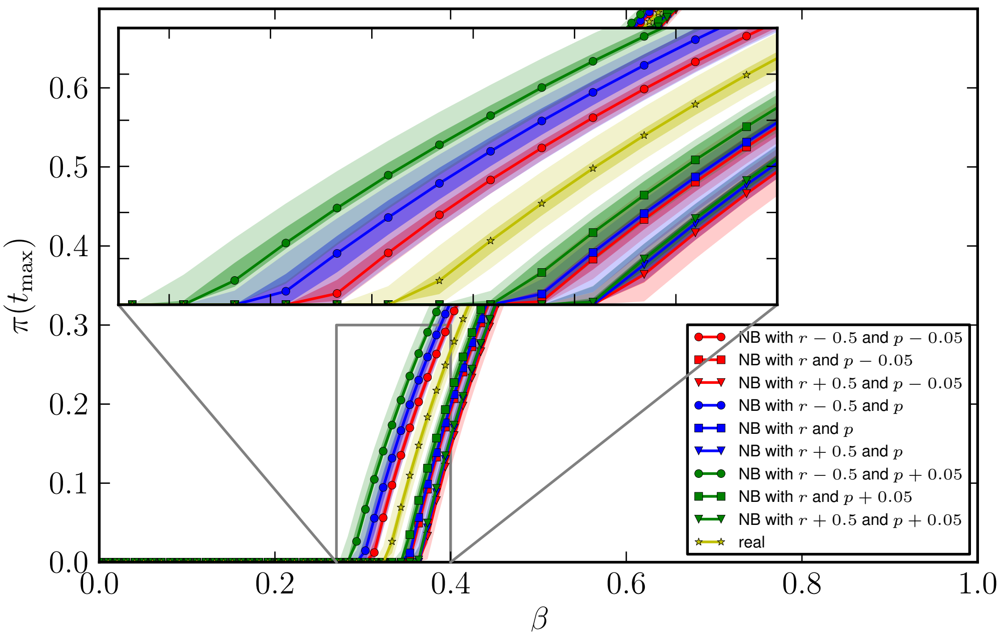

Figure 2. Median (line), interquartile (darker areas) and 95% confidence intervals (lighter areas) of the final prevalence as a function of the transmission probability. Ticks burdens are described by NB distribution with different parameters (see legend). In particular, we explore the sensitivity of this function to variations in r and p, which represent the best fit parameters on the empirical data. The final prevalence obtained with tick burdens sampled from the empirical distribution is also plotted as benchmark.

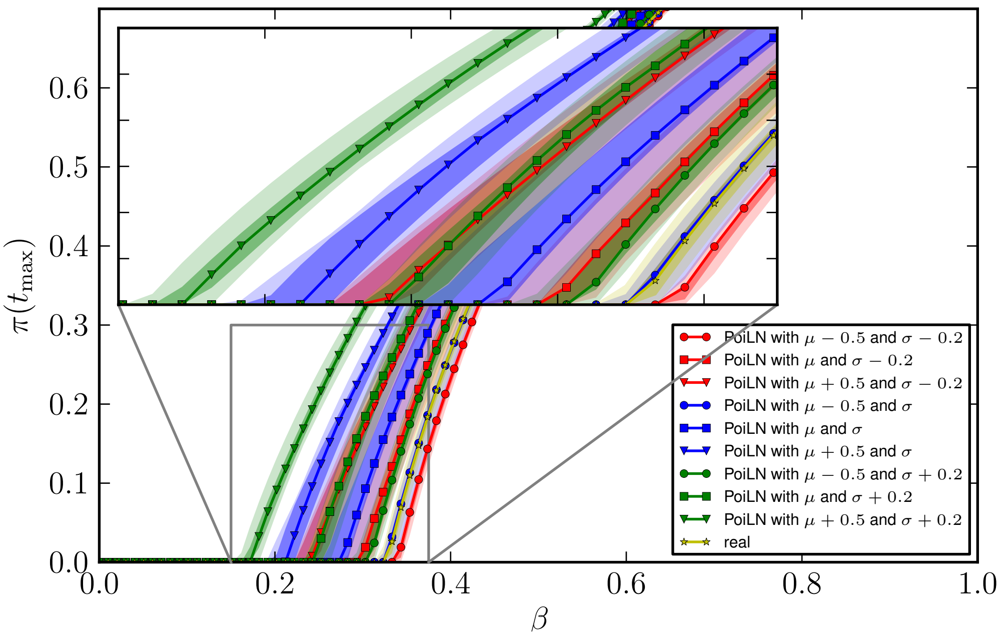

Figure 3. Median (line), interquartile (darker areas) and 95% confidence intervals (lighter areas) of the final prevalence as a function of the transmission probability. Ticks burdens are described by PoiLN distribution with different couple of parameters. In particular, we explore the sensitivity of this curve to variations of μ and σ, the best fit parameters on the empirical data. The final prevalence obtained with tick burdens sampled from the empirical distribution is also plotted as benchmark.
