# Supplementary material for: Pattern of Tick Aggregation on Mice: Larger Than Expected Distribution Tail Enhances the Spread of Tick-Borne Pathogens
Source: PLoS Comput Biol. 2014 Nov 13;10(11):e1003931. doi: 10.1371/journal.pcbi.1003931 (PMC4230730; doi:10.1371/journal.pcbi.1003931)
Supplement: Text S2 — Sensitivity analysis of f , fraction of nymphs among ticks. (PDF) [file pcbi.1003931.s002.pdf]

Pattern of tick aggregation on mice: larger than expected distribution tail enhances the spread of tick-borne pathogens: Supporting Informations S2

Luca Ferreri*, Mario Giacobini, Paolo Bajardi, Luigi Bertolotti, Luca Bolzoni, Valentina Tagliapietra, Annapaola Rizzoli, Roberto Rosà
∗ E-mail: luca.ferreri@unito.it

S2 - Sensitivity Analysis of f, Fraction of Nymphs among Ticks

In this section we explored the effect of different fractions, f, of nymphs overall the total number of ticks on epidemic spreading. By exploring $f = 2\%$ in main text, $f = 5\%$ in Figure 1 and $f = 10\%$ in Figure 2 we conclude that the larger the f, and the larger the probability of invasion of the pathogen. Moreover, it is worth to stress out that for different values of f epidemic curves qualitatively do not change and in particular the order of epidemic thresholds is maintained.

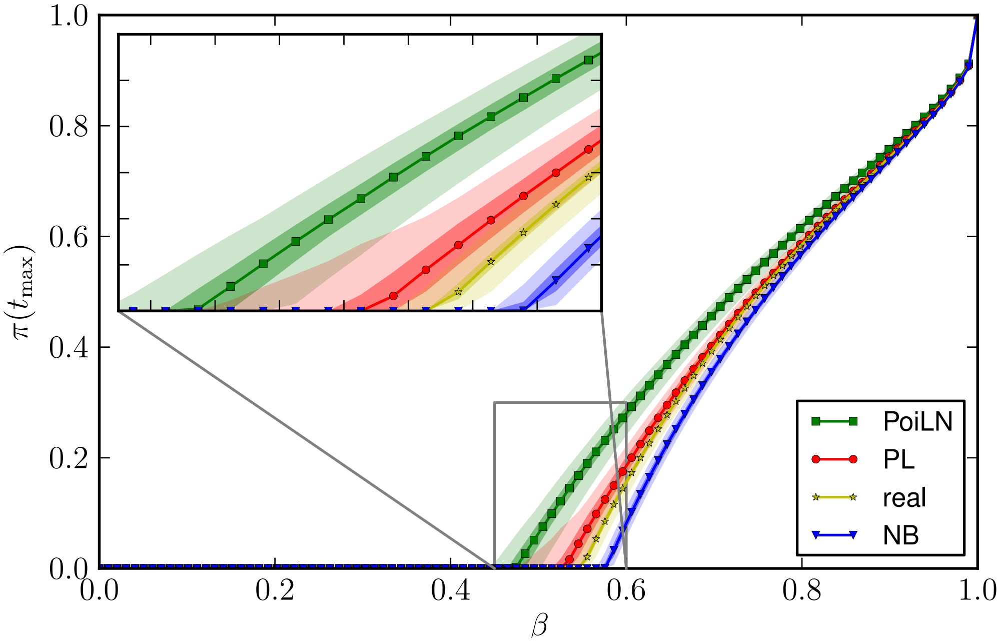

Figure 1. Median (lines), interquartile (darker areas) and 95% confidence intervals (lighter areas) of the final prevalence as a function of the transmission probability. f, fraction of nymphs among ticks is fixed to 5%.

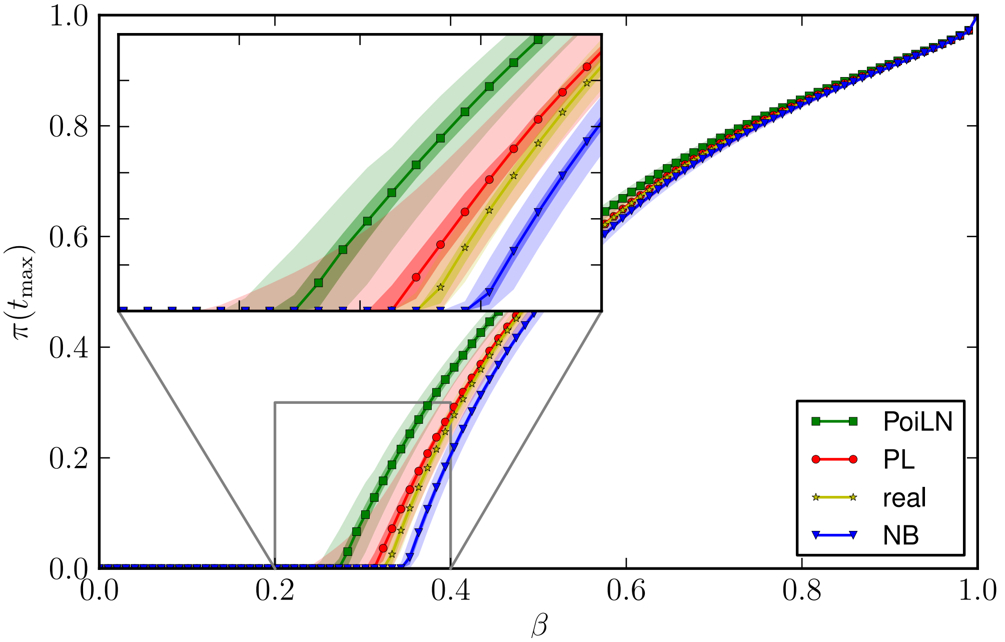

Figure 2. Median (lines), interquartile (darker areas) and 95% confidence intervals (lighter areas) of the final prevalence as a function of the transmission probability. f, fraction of nymphs among ticks is fixed to 10%.
